# Digitalization of Intervention Delivery and Its Impact on the Effects of Interventions for Mental Well-Being in Higher Education Students: Systematic Review and Meta-Analysis Protocol

**DOI:** 10.2196/88458

**Published:** 2026-07-03

**Authors:** Kristoffer Walsund, Daniel Masterson, Rajna Knez

**Affiliations:** 1 School of Health Sciences University of Skövde Skövde, Västra Götaland Sweden; 2 School of Bioscience University of Skövde Skövde, Västra Götaland Sweden; 3 Skaraborg Hospital Skövde, Västra Götaland Sweden

**Keywords:** mental well-being, subjective well-being, positive affect, life satisfaction, mental health promotion, student mental health, higher education, digital mental health

## Abstract

**Background:**

Delivery of interventions within student mental health services has undergone considerable digital transformation in recent years. Traditional face-to-face meetings are being substituted with autonomous digital tools with evident advantages in terms of accessibility and scalability. Despite an increasing array of digital options, there is also a growing recognition that digital tools offer limited effectiveness without some degree of human support. For example, for mental well-being, completely digitally delivered interventions show approximately half the effect sizes of interventions delivered in a traditional format. Blended forms of delivery that use both digital advantages and recognized effects of human contact are therefore promising. Hitherto, the effects of blended delivery have not been evaluated for mental well-being. Hence, investigating how digitalization in intervention delivery impacts intervention effects on mental well-being is important. This is especially relevant among emerging adults enrolled in higher education, going through a critical, transformative life phase.

**Objective:**

This systematic review and meta-analysis will primarily aim to investigate differences in effect due to the degree of digitalization in the mode of delivery of interventions on mental well-being among higher education students.

**Methods:**

This work will adhere to the Cochrane Collaboration methodology, and results will be reported according to PRISMA (Preferred Reporting Items for Systematic Reviews and Meta-Analyses) guidelines. A systematic literature search will be conducted across 9 databases (Scopus, MEDLINE, PubMed, PsycINFO, ERIC, CINAHL, Web of Science, Cochrane, and International Clinical Trials Registry Platform). The population, intervention, comparator, and outcome framework will inform both the search strategy and eligibility criteria. For inclusion, studies should be randomized controlled trials investigating the effect of individually delivered interventions on positive affect or life satisfaction among mentally healthy higher education students aged 18 to 29 years. Studies will be independently screened, and data will be extracted, including the standardized mean difference as the effect measure. Risk of bias assessment will be conducted using the Cochrane Risk of Bias 2 instrument. The Hartung-Knapp-Sidik-Jonkman method for random effects meta-analysis will be applied, and study biases will be analyzed by funnel-plot assessment and Egger test. Finally, certainty of evidence for positive affect and life satisfaction will be assessed using the Grading of Recommendations Assessment, Development, and Evaluation approach. Results will be presented in a summary of findings table.

**Results:**

The search was finalized in March 2026, generating 6603 records after duplicate removal. Study selection and data extraction were conducted during April 2026, resulting in 41 eligible studies. Risk of bias assessment, data analysis, and manuscript preparation are planned before submission for peer review in August 2026.

**Conclusions:**

The principal findings of this study will highlight differences in effect due to the mode of delivery of interventions on mental well-being among higher education students. This will be relevant for the management of student mental health promotion services.

**Trial Registration:**

PROSPERO CRD420251131950; https://www.crd.york.ac.uk/PROSPERO/view/CRD420251131950

**International Registered Report Identifier (IRRID):**

PRR1-10.2196/88458

## Introduction

### Digitalization of Intervention Delivery

Interventions within the mental health area encompass practices aimed at maintaining or improving mental health functioning or conditions [1]. The traditional mode of delivery of mental health interventions at the individual level is that a specialist meets an individual in-person. Even though this mode of delivery is proven to be adjunctively effective for important mental health outcomes [2,3], it is also associated with crucial limitations such as low accessibility, high costs, low scalability, high thresholds, and inferior management of resources [4]. Consequently, there is great interest within the area of mental health to identify viable solutions to circumvent such limitations, and the digitalization of intervention delivery stands out as a particularly promising avenue [5].

As technology advances rapidly, there has been a fast-forward digital revolution within the mental health area, which has generally gone from establishing synchronous telehealth to launching more autonomous innovations independent of person-to-person communication [6]. Contemporary digital devices used for intervention delivery include mobile phones, personal computers, wearable devices, extended reality devices, smart speaker devices, game consoles, and robots. Using these technologies, interventions may be delivered through websites, apps, email, SMS text messaging, push notifications, audio or video conversations, chatbots, and artificial intelligence conversational agents [7]. As such, there is a vast and increasing array of digital options to be used for the delivery of interventions to individuals.

### Mental Health Promotion

Mental health is an umbrella term capturing both mental ill-health and mental well-being as 2 related, but also separate, dimensions [8,9]. As such, mental health promotion can imply the alleviation of mental ill-health or the strengthening of mental well-being [10]. The latter, as a proactive rather than reactive approach, has been researched extensively in later years, and the overall effect of interventions on mental well-being demonstrates small to medium effect sizes [11-14]. Moreover, the relationship between mental well-being and mental ill-health has been thoroughly investigated. When mental well-being is increased, mental ill-health generally is reduced [15,16]. High mental well-being is also linked to a lower likelihood of future mental ill-health [17,18], and conversely, low mental well-being is associated with a higher risk of future mental ill-health [19,20]. Most importantly, this implies that (1) it is possible to strengthen individual mental well-being and (2) it is a viable proactive mental health promotion strategy.

Unfolding mental well-being, the Aristotelian distinction of hedonism and eudaimonism is commonly used by scholars [21,22]. Despite empirical support for both perspectives as being independent but related [23,24], existing research on mental well-being is overrepresented by the hedonic well-being tradition [25], which in turn is most commonly conceptualized in terms of subjective well-being (SWB) [26]. SWB, a multidimensional construct, consists of 2 affective components (ie, experienced positive affect and negative affect) and 1 cognitive component (ie, evaluated life-satisfaction) [27]. Despite some critique (eg, disregarding low-arousing positive affects [28], neglecting moral appropriateness of negative affect [29], and ecological validity of life satisfaction [30]), there is still strong meta-analytic support for the generalizability and robustness of the hierarchical structure of SWB and its subdimensions [31]. Furthermore, SWB is the most widely used outcome within mental well-being intervention research [11,32].

In terms of mental health promotion in general and digitalization of intervention delivery, there is a growing recognition that digital tools offer limited effectiveness without some degree of human support [33]. Concentrating on mental well-being (ie, SWB), systematic compilation of evidence on intervention effectiveness has emerged more recently. Recent reviews have found that entirely digitally delivered interventions yield smaller effect sizes (approximately half the effect) compared with traditional nondigitally delivered interventions [34-36]. However, the blended form of delivery, which uses both digital advantages as well as recognized effects of human contact, has hitherto not been evaluated in relation to mental well-being. Considering mental ill-health outcomes, such as depression, this blended mode of delivery has been shown to generate larger effect sizes [37,38]. Therefore, exploring the effects of interventions on mental well-being based on the degree of digitalization in the mode of delivery, including blended forms of delivery, represents a frontier for the future of mental health promotion.

### Student Mental Health Promotion

One relevant arena for studying the effectiveness of mental health promotion initiatives is student mental health services. Emerging adulthood, the life stage between adolescence and adulthood, is a distinctive developmental period of life characterized by identity exploration, career and relationship instability, and heightened independence [39]. Individuals in this age group, normally specified as 18 to 29 years of age, are identified as more vulnerable to mental ill-health than the general adult population, especially if enrolled in higher education [40]. For example, recent large-scale surveys show that approximately two-thirds of university students experience mental ill-health during their studies [41] and that only 1 out of 10 report high levels of mental well-being [42]. Hence, emerging adults in higher education are recognized as a population with significant mental health support needs [40,43]. Also, research has demonstrated that students themselves wish for mental health support in terms of proactive approaches [44]. Students are furthermore a group that tends to take greater use of mental health services when there are more options available [45], and students commonly report insufficient knowledge of accessible resources as a barrier to active use of mental health support [46]. Moreover, in relation to interventions within the student mental health area, mental well-being promotive interventions are understudied relative to interventions focused on mental ill-health outcomes [47]. Taken together, this illustrates the relevance of more research on proactive initiatives and advancements within the field of student mental health promotion.

Considering the degree of digitalization in intervention delivery, students have been shown to value and desire a variety of modes of contact with student mental health services [44]. However, there is still an expressed need for more research to explore intervention effectiveness based on the digitalization of intervention delivery within the student mental health area [48]. Searching databases as well as the PROSPERO register, a total of 7 reviews of relevance were identified. In essence, the vast majority of previous reviews have investigated the effects on mental ill-health outcomes rather than mental well-being [48-53]. Only 1 previous review presented results on the effect of digital interventions on mental well-being among higher education students [54]. Based on 11 studies, a small, significant effect size was found for digital interventions on the mental well-being of students as compared with controls. The risk of bias in included studies was considered high, and mental well-being was represented by 7 different operationalizations in the included studies. Also, no further analyses were performed in relation to differences between digital modalities or intervention characteristics. Considering intervention categories (ie, general types or themes of interventions), only 1 subgroup analysis was performed in the study, capturing a significant moderate effect of acceptance and commitment therapy-based interventions. Summarizing, this underscores the importance of exploring the effects of interventions on mental well-being in isolation within the context of promotive student mental health services. Especially, it gives reasons to investigate the impact of degrees of digitalization in intervention delivery as well as to study the contrasts in effect between different categories of interventions.

### Aim and Objectives

The primary aim of this review is to examine whether and how the effects of interventions on mental well-being vary by the degree of digitalization in the mode of delivery among higher education students. In more detail, it will contrast 3 delivery modes—*entirely digital* (interventions delivered by fully automated digital tools with no human contact), *partly digital* (interventions delivered partly through human contact and partly with assistance of digital tools), and *nondigital* (interventions delivered only through human contact with, for example, health specialists). As a secondary aim, this review will explore differences in the effect on mental well-being of different categories of interventions. This implies that general types or themes of interventions will be clustered and subanalyzed. In relation to both aims, mental well-being will be operationalized as positive affect and life satisfaction. Hence, the two main research questions in this review are:

1. What are the differences in effect due to the level of digitalization in the mode of delivery of interventions on positive affect and life satisfaction, respectively, among higher education students?
2. What are the differences in effect between different categories of interventions on positive affect and life satisfaction, respectively, among higher education students?

## Methods

### Design

This systematic review and meta-analysis will be guided by the Cochrane Handbook for Systematic Reviews of Interventions [55], will adhere to PRISMA (Preferred Reporting Items for Systematic Reviews and Meta-Analyses) reporting guidelines [56], and is preregistered in the PROSPERO database. A completed PRISMA-P (Preferred Reporting Items for Systematic Review and Meta-Analysis Protocols) checklist [57] for the reporting of this protocol is available in Multimedia Appendix 1. The study will overall be composed of a systematic literature search, study selection, data extraction, quality assessment, data analysis, and synthesis. Throughout the study, the population, intervention, comparator, and outcome (PICO) structure, as informed by the main research questions, will guide the work.

### Definition of PICO

The scope of this review will be demarcated by definitions of respective PICO elements as follows.

#### Population

The target population will be students enrolled in higher education between the ages of 18 and 29 years who are considered mentally healthy. Higher education will encompass education at the postsecondary level delivered by universities, colleges, and other tertiary institutions in on-campus, online, or blended formats. In general, this will capture young or emerging adults who are in a multitransformative life situation. In relation to mental health, in this review, mentally healthy will imply that individuals are not diagnosed with any mental disorder or other chronic mental health condition. This will be determined based on how the participant’s condition is reported in individual records, either based on sampling criteria or how the results of baseline mental health are interpreted by the original authors. If mental ill-health is not measured or screened for in individual studies, participants will be considered as mentally healthy. Individuals with nonclinical levels of mental distress, such as mild stress, anxiety, low mood, depressive symptoms, or sadness, will be considered part of the target population. Participants may also be relatives or caregivers of ill people, and they may have been previously mentally ill themselves but have recovered. There will be no other explicit demographic limitations.

#### Intervention

This review will include interventions intended to promote mental well-being at the individual level. Interventions may be framed as a practice, training, program, activity, exercise, therapy, counseling, workshop, course, or similar. Singular interventions, in the sense that they represent a distinct theme (eg, gratitude, mindfulness, and physical exercise), as well as multicomponent or step-by-step programs, will be part of this review. Crucially, they should be intended to affect either positive affect or life satisfaction or both aspects. Interventions may have been delivered in any mode and in any context as long as they have been delivered individually (ie, not in a group setting). The rationale for not including group-delivered interventions is that this research is focused on factors related to the intimate alliance between the deliverer and the person. This therapeutic relational bond is a well-established adjunct factor of intervention effect [3] and key to the study of possibilities and limitations of technological advancements related to the delivery of interventions [58,59].

#### Comparator

To be of relevance for this review, studies need to have applied a design in which participants were randomly assigned to 1 or more intervention arms versus 1 or more control or comparison arms. The comparison arm may not consist of a different active intervention. Acceptable comparators are *assessment-only* (the control arm receives only the measurement procedures, administered at intervals comparable with the intervention arm), *waiting list* (the control arm receives the same measurement procedures and is offered the intervention after completion of the measurement phase, *passive control* (the control arm completes an activity of similar duration to the intervention arm but one that is not designed to isolate the intervention’s active component), or *active control* (the control arm completes an activity of similar duration that is designed to isolate the active component of the intervention; all other elements are held constant).

#### Outcome

The primary outcomes will be the differences between the intervention group and the comparator group in changes from pre to post and follow-up measures (ie, interaction effects) of mental well-being. Mental well-being in this research will be defined as SWB [26]. More specifically, it will be represented by SWB’s promotion-related subcomponents—positive affect and life satisfaction. The rationale for not including the traditional SWB subcomponent of negative affect is to exclusively explore more strictly proactive mental well-being initiatives. Research on approaches intended to alleviate negative mental experiences is in abundance; thus, more knowledge on strengthening focused interventions is called for within the mental health area [60]. Concerning positive affect, both high- and low-arousing affective states or experiences will be considered. In terms of life satisfaction, evaluative judgments about one’s current life as a whole, as well as in relation to different life domains, will be of interest. Outcomes shall have been measured with validated introspective questionnaires.

### Search Strategy

The overall search strategy for this review will be to identify as many relevant studies as possible that match the scope as outlined by the PICO. The inclusiveness and precision of the search will be balanced in regard to available resources in order to secure a manageable project.

The search strategy was developed by the first author (KW) together with a professional research librarian and reviewed by the research team as well as stakeholders from the local university student health service. This process generally included the selection of databases and development of search content (blocks and terms), search techniques (fields and operators), and search adaptation (database interface adapted queries). The process also involved piloting and evaluating search queries. The Peer Review of Electronic Search Strategies (PRESS) Evidence-based Checklist [61] applied to the MEDLINE search was used as guidance for quality assurance and final revisions of the search strategy.

The date of the final search will be explicitly stated. Alerts will be set for all searches and screened up until the submission of the final manuscript. Depending on whether quantitative synthesis has been performed or not, studies meeting the eligibility criteria will be either included or else mentioned as “study awaiting assessment” in the supplementary material.

### Information Sources

The data sources for this review will be peer-reviewed journal articles presenting original studies, as sourced via databases Scopus, MEDLINE (EBSCOhost), PubMed, PsycINFO (ProQuest), ERIC (EBSCOhost), CINAHL (EBSCOhost), Web of Science, Cochrane CENTRAL, as well as International Clinical Trials Registry Platform. Additionally, reference lists in included studies and relevant reviews identified will be checked for eligible studies. There will be no set time constraint as to when studies should have been published; rather, this will only be limited by the time frames available in selected databases. PubMed will only be used in order to complement the MEDLINE search with potential records not yet indexed in MEDLINE, and therefore, a subset field search for records not published in MEDLINE will be used in PubMed.

### Search Terms

Relevant search terms for mental well-being were developed over an extended period (years) as the first author (KW) has actively followed international research on mental well-being, especially focusing on mental well-being conceptualizations and effects of interventions. For the specific population or context (ie, higher education students and mental health services), several recent bibliometric mapping studies were reviewed to identify central search terms. With respect to study design and general labels of interventions, a number of recently published reviews similar to the current review were screened to identify relevant terms. In addition, potentially relevant terms as well as coverage of selected terms were explored and evaluated using the thesauri of the planned databases. Finally, generative artificial intelligence (ChatGPT 5.2 [OpenAI] and Scopus AI [Elsevier]) was used to explore possibly unobserved terms, and no additional search terms were added or refined out of this step. The final set of search terms was established through systematic pilot testing and evaluation, primarily in the MEDLINE and Scopus databases.

The search query will contain 4 main blocks of search terms, both text words and standardized subject terms (eg, Medical Subject Headings terms), guided by PICO. Text words will be identical across all databases and are presented in Table 1. Standardized subject terms will be identified and applied adaptively according to each database’s indexing. In many cases, standardized subject terms did not add to the existing text words in pilot test searches; thus, standardized subject terms will be used in moderation only when they add substantially to the number of records identified. The subject terms to be used are presented in Multimedia Appendix 2 within the detailed search queries to be used in the respective database. No filters or search limitations will be applied in any database. The completed PRISMA-S (Preferred Reporting Items for Systematic Review and Meta-Analysis Search) checklist [62] is available in Multimedia Appendix 3.

**Table 1:** Search query text words as structured by population, intervention, comparator, and outcome.

PICO^a^-domain	Field	Text words
Population	Title, abstract, and keywords	(student* OR universit* OR college* OR “higher education” OR campus OR tertiar*)
Intervention	Title, abstract, and keywords	(interven* OR practic* OR train* OR activit* OR exercis* OR program* OR promot* OR therap* OR counsel* OR workshop* OR course* OR support)
Comparator	Title, abstract, and keywords	(random* [proximity operator within 3 words] (trial* OR assign* OR allocat*))
Outcome	Title, abstract, and keywords	(happiness OR happy OR flourish* OR “positive mental health” OR joy* OR ((subjective OR mental OR positive OR psych* OR personal*) [proximity operator within 3 words] (well-being OR wellbeing OR wellness OR satisf*)) OR ((life OR domain*) [proximity operator within 3 words] (satisf* OR evalua* OR appraisal)) OR (positive [proximity operator within 3 words] (affect* OR emotion* OR mood* OR feeling*))

^a^PICO: population, intervention, comparator, and outcome.

### Study Selection

All citations identified by the systematic literature search in all databases will be downloaded and subsequently uploaded into the Covidence software (Veritas Health Innovation Ltd) [63], where duplicates will be initially removed. A minimum of 2 authors will then independently screen the titles and abstracts of all records to identify studies eligible for inclusion based on the set inclusion criteria. Each criterion will be transformed into a straightforward yes-or-no question to be asked by the reviewers when conducting the first-stage screening, as presented in Multimedia Appendix 4. In case of uncertainty, there will also be a maybe option for each screening question. The screening process will initially be piloted in order to establish consistency across reviewers. Disagreement about whether to include a specific record or not will be resolved by discussion of each study, with an option to consult an extra reviewer. If disagreement or uncertainty still exists, the full-text version of the records will be reviewed and discussed until consensus is reached.

In the next stage, full-text versions of all studies identified as eligible for inclusion will be sought. At least 2 authors will then independently perform a full-text screening of all studies and exclude ineligible studies while also indicating reasons for exclusion. Decisions at this stage will be based on the detailed PICO as well as the exclusion criteria. Disagreement or uncertainty between review authors will again be resolved through discussion, with the same option to consult an extra reviewer, until consensus is reached. Interrater reliability will be calculated for both stages of screening. All screening will be performed in the Covidence software, and a PRISMA flowchart will be constructed to report the step-by-step study selection process. When full-text versions are not available due to a paywall or similar, the corresponding author (KW) will be contacted. If no response within 2 weeks, the record will be excluded.

### Inclusion Criteria

Both stages of screening for eligible studies will be performed based on a set of inclusion criteria informed by the PICO domains. In the first stage of title and abstract screening, the inclusion criteria will serve as justification for inclusion to further full-text evaluation. In the second stage of screening full-text manuscripts, the inclusion criteria will be applied and informed by the more nuanced and detailed description of each PICO domain.

The first inclusion criterion relates to the population. This review aims to add to existing knowledge in the area of student mental health promotion. The focus is on proactive approaches that strengthen mental well-being and build resilience to potential future mental ill-health. Accordingly, the study participants should be (1) students in higher education aged 18-29 years, and (2) considered mentally healthy.

The second inclusion criterion concerns the interventions. This review aims to evaluate the differences in the effect of interventions based on the degree of digitalization in the mode of delivery. Digitally delivered interventions are typically individually delivered (ie, not to groups), so in order to be able to perform a meaningful comparison, only studies where interventions are delivered individually will be included.

The third inclusion criterion is focused on the comparator and related study design. Studies will be included only if they use a randomized experimental design in which participants are assigned to one or more intervention arms versus one or more control or comparison arms. The comparison arm may not consist of a different intervention, and acceptable comparators are assessment-only, waiting list, passive control, and active control. Treatment-as-usual (TAU) comparators are often accepted in reviews of this type. In this review, TAU comparators will be treated with caution since they, by definition, indicate that participants are undergoing some form of treatment and thus cannot reasonably be considered fully healthy. Hence, studies applying the TAU comparator will not be excluded by default, but are likely not to be included due to the first inclusion criterion. Moreover, studies using nonstandard randomized designs such as cluster-randomized trials or crossover trials will not be included.

The fourth inclusion criterion captures the outcomes. This review will adopt a strict mental well-being focus. There is a clear overrepresentation of research on mental ill-health within the broader field of mental health, whereas the positive dimension—mental well-being—has been studied far less. Consequently, there is an explicitly stated need for additional research on mental well-being, including compiling the evidence on the effects of interventions and potential moderators. Therefore, only studies that present the effect of interventions on SWB components—positive affect, life satisfaction, or both—as either primary or secondary outcome will be included. The affective component of SWB is traditionally described as the relative frequency of positive and negative affective experiences. In this review, only positive affect will be examined. The rationale is that negatively valenced experiences (eg, distress and anxiety) have already been extensively investigated within the traditional, more pathogenic mental health paradigm. This review aims to contribute to knowledge on more strictly proactive and promotive approaches focused on strengthening mental well-being; hence, positive affect will be considered in isolation.

### Exclusion Criteria

Studies may fulfill the inclusion criteria in the first stage screening, and also match all PICO domains in the second stage screening, but still not be eligible for inclusion. Justification for such decisions will be based on the following exclusion criteria, which will be applied during the second stage full-text screening.

The first exclusion criterion concerns details in information about the mode of delivery of interventions. The primary aim of this review is to contrast the effects of interventions across delivery modes defined by their level of digitalization. Accordingly, all studies will be classified once the record screening has been completed as entirely digital, partly digital, or nondigital. To allow reliable classification, the mode of delivery must be explicitly and unambiguously described in the published study report or its supplementary material. In more detail, the intervention design must be reported in sufficient depth to be fully replicable in regard to which elements were digital and which were non-digital.

Second, only full-text records will be included to enable a meaningful and unbiased review and synthesis. This criterion also excludes conference papers and other similarly brief study reports. In addition, only studies published in peer-reviewed scientific journals will be included; original studies reported in textbooks or similar non–peer-reviewed sources are excluded.

### Data Extraction

Data will be extracted independently by a minimum of 2 authors using the Covidence software, and at least 1 other author will independently verify the extracted data. The general strategy for data extraction will be to both extract data from the study, which will inform the evidence synthesis, and data about the study that will inform the risk of bias assessment. A list of all included studies with all extracted data will be provided.

Extracted information from respective studies will include details about participants, intervention, comparator, and outcome. The Covidence software will enable extracted data to be directly and jointly compiled into a single data collection form. The intended structure and content of the data collection form are presented in Multimedia Appendix 5. Any disagreements or uncertainties, either between the data extractors or when the other author or authors verify the extracted data, will be resolved through discussion. Potential discrepancies will be noted and presented as part of the supplementary material. In case a performed study is presented in multiple reports, the information or data from each report will be collected separately and then combined to represent one single report in the data collection form. In relation to missing data or uncertainty concerning statistics, the authors of the original study will be contacted. If no response is received within 2 weeks or if the response does not bring clarity, the data will be considered as not available. The full data extraction process will first be piloted in order to establish a systematic and consistent practice. On a final note, more general information, such as in which country the study was performed, year of publication, and so on, will also be extracted from each study.

### Risk of Bias Assessment

As mentioned, extracted information *about* the respective study will inform the assessment of risk of bias. This information will follow the 5 domains of the Cochrane Risk of Bias 2 (RoB2) assessment tool [64] and include information concerning the randomization process, deviations from intended interventions, missing outcome data, outcome measurement, and selection of reported results (for more details, refer to Multimedia Appendix 4). The intention-to-treat effect will be of principal interest for the risk of bias assessment and will be conducted in relation to the interaction effect in each study of the difference between groups in changes from pre- to postmeasures of positive affect or life satisfaction or both.

At least 2 authors will independently assess the risk of bias in each study using the RoB2. The procedure will be piloted to achieve a systematic assessment. Disagreements in judgments will be resolved through discussion. Summary judgments about the overall risk of bias for results on positive affect or life satisfaction or both in each study will be categorized as either low risk of bias, some concerns, or high risk of bias. Risk of bias judgements, both domain-wise and overall, will be presented in forest plots alongside the results of each study included in meta-analyses. If applicable, additional sensitivity subgroup analyses excluding studies with a high risk of bias will be conducted.

### Data Analysis and Synthesis

Data synthesis will be conducted in terms of an initial preparation phase including (1) summarizing the included studies, (2) determining which studies are feasible to be grouped within each comparison, (3) determining what data are available for synthesis, (4) determining if modification of the planned analyses is necessary, and (5) synthesizing the characteristics of the studies contributing to each comparison. The next phase will then involve statistical synthesis as well as interpretation and description of the results. If there is a lack of data amenable to meta-analysis, only narrative synthesis will be performed based on the synthesis without meta-analysis guidelines [65].

In terms of grouping studies for comparison, information concerning the mode of delivery and intervention category will be used. Mode of delivery will consist of the 3 prespecified groups of entirely, partly, and nondigital delivery formats as described earlier in this protocol. In case it is found possible and meaningful to group studies even more granularly in terms of mode of delivery (eg, dividing the partly digital group into relevant subgroups), this will be considered in regard to space (same-distributed) and time (synchronous-asynchronous) of the human interaction. The intervention categories groups will evolve during the process of screening, extracting data, and summarizing the included studies. The intention to perform subgroup meta-analyses on intervention categories will also serve as a guiding principle when grouping studies based on intervention theme; thus, merging of categories with too few studies into broader categories might be needed. On beforehand, a preliminary list of potentially relevant categories includes physical activity, gratitude, savoring, optimism, nature, meditation, mindfulness, altruism, forgiveness, cognitive reappraisal, light therapy, laughter, arts, character strengths, compassion, self-compassion, social interventions, goal-setting, acceptance, self-control, breathing, values, growth mindset, creativity, humor, self-reassurance, body awareness, prayer, yoga, relaxation, expressive writing, behavior activation, reminiscence, positive reframing etcetera.

Included studies will most likely report the outcome using different scales, and therefore, the standardized mean difference (SMD) will presumably be used as an effect measure. Furthermore, it is reasonable to assume that the different studies will be estimating different, yet related, intervention effects. Based on this conceptual assumption, a random-effects meta-analysis will most likely be relevant to apply. Statistical heterogeneity across pooled studies intended for meta-analysis is assumed to exist. Therefore, if a reasonable number of studies are possible to include in meta-analysis (eg, more than 10) in order to assume normal distribution for the effect across studies, prediction intervals will be presented in order to transparently express the amount of heterogeneity. Most likely, the Hartung-Knapp-Sidik-Jonkman method will be applied for the meta-analysis on differences in effect due to mode of delivery. This method has been demonstrated to produce more realistic confidence intervals, reducing false-positive findings, especially when studies are heterogeneous, than the standard DerSimonian-Laird method [66]. The same method will most likely be applied for meta-analysis on differences in effect between categories of interventions, but if found feasible based on the characteristics of included studies, a network meta-analysis approach will also be considered.

Additionally, on a general note, the possibility of using baseline outcome measurements as a covariate in a regression model will also be considered in order to minimize bias in estimates of intervention effect due to baseline differences. Moreover, categorical moderator analyses will be performed for other demographic, cultural, or study characteristic factors of relevance. Study biases will be assessed by visual examination of funnel plots and by the Egger test. Also, sensitivity subgroup analyses based on risk of bias assessments will be conducted. Analyses will most likely be performed in IBM SPSS.

SMD effect sizes will be reported in combination with their 95% CIs and associated *P* values. SMD of 0.2 will be interpreted as a small effect, 0.5 as a moderate effect, and 0.8 as a large effect. A *P* value of .05 will need to be met in order for observed effects to be counted as statistically significant.

Finally, the overall effect on mental well-being and certainty of evidence for all interventions, independent of level of digitalization in mode of delivery, will be assessed using the Grading of Recommendations Assessment, Development, and Evaluation approach [67]. The GRADEpro GDT tool (Evidence Prime) [68] will be used, and critical outcomes will be positive affect and life satisfaction. Results will be presented in a summary of findings table alongside the provision of rationales for the grading of evidence.

## Results

The systematic literature search was performed in January 2026 and updated with results from search alerts on March 13, 2026. A total of 13,212 records were identified, and after removal of duplicates 6603 records were valid for title and abstract screening. The title and abstract screening resulted in 328 records being eligible for full-text review. After full-text review, a total of 41 records fulfilled all inclusion criteria and were decided to be eligible for inclusion in the study. Data were extracted from all included studies during April 2026. Taken together, the literature search, study selection, and data extraction were all performed without any major deviations from the original protocol.

The risk of bias assessment was started during May 2026 and will be followed by data analysis in June 2026. The final manuscript is being written simultaneously as the work progresses and is planned to be submitted for peer review in August 2026. In case of needing to amend this protocol, the date and description of the respective change, together with the rationale, will be presented in the Methods section of the final manuscript. Changes will not be incorporated into the protocol.

## Discussion

The principal findings of this review will highlight whether and how the effects of interventions on mental well-being among higher education students vary by the degree of digitalization in the mode of delivery. Unlike any previous meta-analysis, it will compile and contrast the effects on mental well-being of entirely digital, partly digital, and nondigital delivery of interventions. In the area of mental well-being, entirely digitally delivered interventions have been demonstrated to yield smaller effect sizes in comparison with nondigitally delivered interventions [34,35,69]. However, the effects of partly digitally delivered interventions on mental well-being are yet to be evaluated. Concerning depression as a related outcome, human-guided digital delivery (ie, partly digital) has interestingly been demonstrated to produce twice as strong effect sizes compared with entirely digital delivery [37]. Hence, valuable insights might be concealed in the intermediate terrain of partly digital delivery.

This review will also generate knowledge on the effects of different categories of interventions on mental well-being among higher education students. In terms of the general mentally healthy adult population, there are several meta-analyses giving support for causal effects of a variety of interventions on mental well-being (eg, studies by van Agteren et al [11], Carr et al [12], Donaldson et al [14], Koydemir et al [34], Hendriks et al [70], Carr et al [71], Blodgett et al [72], Kraiss et al [73], and van Dierendonck and Lam [74]). Furthermore, withholding focus on the same general population, there is meta-analytical support for a range of categories of interventions on more precise components of mental well-being, such as positive affect and life satisfaction (eg, studies by Buecker et al [75], Galante et al [76], Eberth and Sedlmeier [77], Schutte and Malouff [78], Carrillo et al [79], Akhtar and Barlow [80], Gaekwad et al [81], and Bacaro et al [82]). Nevertheless, when further narrowing in on higher education students as a target group, meta-analytical studies are scarce. Thus, the findings of this review are anticipated to constitute an important contribution to the promotion of student mental well-being.

The fairly narrow focus of this review, together with the application of relatively strict eligibility criteria, may be perceived as both strengths and weaknesses. This set-up will favor internal validity as well as relevance for conducting meta-analysis, while simultaneously limiting generalizability and possibly missing out on aspects of real-world relevance. The tilt toward a narrow conceptualization of mental well-being as SWB’s subcomponents, positive affect, and life satisfaction is motivated by a common critique concerning conceptual confusion within the mental well-being field of study [83]. In relation to generalizability, it will be of particular importance to be meticulous when formulating claims based on the results of this review. Moreover, to ensure practical relevance, the search strategy was discussed during the development phase with mental health professionals from the local university student health service. This contact will also be continuously used for relevance-checking throughout the study.

Building on this, this review will be conducted with the ambition to present an unbiased and detailed summary of evidence. As such, this work will offer new insights and nuanced knowledge considering how and which interventions are most efficiently delivered to students in order to promote and strengthen their mental well-being. This information will be useful to service management for planning and implementing promotive initiatives. The findings may also highlight challenges in need of future attention within the field of study.

## Data Availability

All data generated or analyzed during this study will be included in the published article (and its supplementary information files).

## Appendix

Multimedia Appendix 1 PRISMA-P checklist.

Multimedia Appendix 2 Search queries used for final piloting of planned searches.

Multimedia Appendix 3 PRISMA-S checklist.

Multimedia Appendix 4 Data extraction form.

Multimedia Appendix 5 First stage screening questions.

Multimedia Appendix 6 ChatGPT transcript title reformatting.

## Abbreviations

PICO: population, intervention, comparator, outcome
PRESS: Peer Review of Electronic Search Strategies
PRISMA: Preferred Reporting Items for Systematic Reviews and Meta-Analyses
PRISMA-P: Preferred Reporting Items for Systematic Review and Meta-Analysis Protocols
PRISMA-S: Preferred Reporting Items for Systematic Review and Meta-Analysis Search
ROB2: Cochrane Risk of Bias 2
SMD: standardized mean difference
SWB: subjective well-being
TAU: treatment-as-usual
