# Supplementary material for: Digitalization of Intervention Delivery and Its Impact on the Effects of Interventions for Mental Well-Being in Higher Education Students: Systematic Review and Meta-Analysis Protocol
Source: JMIR Res Protoc. 2026 Jul 3;15:e88458. doi: 10.2196/88458 (PMC13379693; doi:10.2196/88458)
Supplement: Multimedia Appendix 2 [file resprot_v15i1e88458_app2.docx]

# Appendix 2

The number of records presented for each search represents the results of the most recent pilot search before preregistering this protocol in the PROSPERO database. All final pilot searches were performed in the period of October 2025.

| **Information source** | **Search query** | **Records** |
| --- | --- | --- |
| MEDLINE  (Ebsco, Basic search) | XB ((student* OR universit* OR college* OR "higher education" OR campus OR tertiar*) AND (interven* OR practic* OR train* OR activit* OR exercis* OR program* OR promot* OR therap* OR counsel* OR workshop* OR course* OR support) AND ((random* N3 (trial* OR assign* OR allocat*)) OR RCT) AND (happiness OR happy OR flourish* OR "positive mental health" OR ((subjective OR mental OR positive OR psych* OR personal*) N3 ("well-being" OR wellbeing OR wellness OR satisf*)) OR ((life OR domain*) N3 (satisf* OR evalua* OR appraisal)) OR (positive N3 (affect* OR emotion*OR mood* OR feeling*)) OR joy*)) OR SU ((student* OR universit* OR college* OR "higher education" OR campus OR tertiar*) AND (interven* OR practic* OR train* OR activit* OR exercis* OR program* OR promot* OR therap* OR counsel* OR workshop* OR course* OR support) AND ((random* N3 (trial* OR assign* OR allocat*)) OR RCT) AND (happiness OR happy OR flourish* OR "positive mental health" OR ((subjective OR mental OR positive OR psych* OR personal*) N3 ("well-being" OR wellbeing OR wellness OR satisf*)) OR ((life OR domain*) N3 (satisf* OR evalua* OR appraisal)) OR (positive N3 (affect* OR emotion*OR mood* OR feeling*)) OR joy*)) | 1.557 |
| Scopus  (Advanced search) | TITLE-ABS-KEY ((student* OR universit* OR college* OR "higher education" OR campus OR tertiar*) AND (interven* OR practic* OR train* OR activit* OR exercis* OR program* OR promot* OR therap* OR counsel* OR workshop* OR course* OR support) AND ((random* W/3 (trial* OR assign* OR allocat*)) OR RCT) AND (happiness OR happy OR flourish* OR "positive mental health" OR ((subjective OR mental OR positive OR psych*) W/3 (well-being OR wellbeing OR wellness)) OR ((life OR domain*) W/3 (satisf* OR evalua* OR appraisal)) OR (positive W/3 (affect* OR emotion* OR mood* OR feeling*)) OR joy*)) | 2.564 |
| PsychINFO  (Advanced search) | ((student* or universit* or college* or "higher education" or campus or tertiar*) and (interven* or practic* or train* or activit* or exercis* or program* or promot* or therap* or counsel* or workshop* or course* or support) and ((random* adj3 (trial* or assign* or allocat*)) or RCT) and (happiness or happy or flourish* or "positive mental health" or ((subjective or mental or positive or psych* or personal*) adj3 (well-being or wellbeing or wellness or satisf*)) or ((life or domain*) adj3 (satisf* or evalua* or appraisal)) or (positive adj3 (affect* or emotion* or mood* or feeling*)) or joy*)).mp. | 1.302 |
| ERIC  (Ebsco, Basic search) | TI ((student* OR universit* OR college* OR "higher education" OR campus OR tertiar*) AND (interven* OR practic* OR train* OR activit* OR exercis* OR program* OR promot* OR therap* OR counsel* OR workshop* OR course* OR support) AND ((random* N3 (trial* OR assign* OR allocat*)) OR RCT) AND (happiness OR happy OR flourish* OR "positive mental health" OR ((subjective OR mental OR positive OR psych* OR personal*) N3 ("well-being" OR wellbeing OR wellness OR satisf*)) OR ((life OR domain*) N3 (satisf* OR evalua* OR appraisal)) OR (positive N3 (affect* OR emotion* OR mood* OR feeling*)) OR joy*)) OR AB ((student* OR universit* OR college* OR "higher education" OR campus OR tertiar*) AND (interven* OR practic* OR train* OR activit* OR exercis* OR program* OR promot* OR therap* OR counsel* OR workshop* OR course* OR support) AND ((random* N3 (trial* OR assign* OR allocat*)) OR RCT) AND (happiness OR happy OR flourish* OR "positive mental health" OR ((subjective OR mental OR positive OR psych* OR personal*) N3 ("well-being" OR wellbeing OR wellness OR satisf*)) OR ((life OR domain*) N3 (satisf* OR evalua* OR appraisal)) OR (positive N3 (affect* OR emotion* OR mood* OR feeling*)) OR joy*)) OR SU ((student* OR universit* OR college* OR "higher education" OR campus OR tertiar*) AND (interven* OR practic* OR train* OR activit* OR exercis* OR program* OR promot* OR therap* OR counsel* OR workshop* OR course* OR support) AND ((random* N3 (trial* OR assign* OR allocat*)) OR RCT) AND (happiness OR happy OR flourish* OR "positive mental health" OR ((subjective OR mental OR positive OR psych* OR personal*) N3 ("well-being" OR wellbeing OR wellness OR satisf*)) OR ((life OR domain*) N3 (satisf* OR evalua* OR appraisal)) OR (positive N3 (affect* OR emotion* OR mood* OR feeling*)) OR joy*)) | 154 |
| CINAHL  (Ebsco, Basic search) | XB ((student* OR universit* OR college* OR "higher education" OR campus OR tertiar*) AND (interven* OR practic* OR train* OR activit* OR exercis* OR program* OR promot* OR therap* OR counsel* OR workshop* OR course* OR support) AND ((random* N3 (trial* OR assign* OR allocat*)) OR RCT) AND (happiness OR happy OR flourish* OR "positive mental health" OR ((subjective OR mental OR positive OR psych* OR personal*) N3 ("well-being" OR wellbeing OR wellness OR satisf*)) OR ((life OR domain*) N3 (satisf* OR evalua* OR appraisal)) OR (positive N3 (affect* OR emotion* OR mood* OR feeling*)) OR joy*)) OR SU ((student* OR universit* OR college* OR "higher education" OR campus OR tertiar*) AND (interven* OR practic* OR train* OR activit* OR exercis* OR program* OR promot* OR therap* OR counsel* OR workshop* OR course* OR support) AND ((random* N3 (trial* OR assign* OR allocat*)) OR RCT) AND (happiness OR happy OR flourish* OR "positive mental health" OR ((subjective OR mental OR positive OR psych* OR personal*) N3 ("well-being" OR wellbeing OR wellness OR satisf*)) OR ((life OR domain*) N3 (satisf* OR evalua* OR appraisal)) OR (positive N3 (affect* OR emotion* OR mood* or feeling*)) OR joy*)) | 714 |
| Web of Science  (Advanced search query builder) | TS=((student* OR universit* OR college* OR "higher education" OR campus OR tertiar*) AND (interven* OR practic* OR train* OR activit* OR exercis* OR program* OR promot* OR therap* OR counsel* OR workshop* OR course* OR support) AND ((random* NEAR/3 (trial* OR assign* OR allocat*)) OR RCT) AND (happiness OR happy OR flourish* OR "positive mental health" OR ((subjective OR mental OR positive OR psych* OR personal*) NEAR/3 (well-being OR wellbeing OR wellness OR satisf*)) OR ((life OR domain*) NEAR/3 (satisf* OR evalua* OR appraisal)) OR (positive NEAR/3 (affect* OR emotion* OR mood* OR feeling*)) OR joy*)) | 2.074 |
| Cochrane CENTRAL  (Advanced search, Search manager)  Note: The default “search word variations” function was disabled. | ((student* OR universit* OR college* OR "higher education" OR campus OR tertiar*) AND (interven* OR practic* OR train* OR activit* OR exercis* OR program* OR promot* OR therap* OR counsel* OR workshop* OR course* OR support) AND ((random* NEAR/3 (trial* OR assign* OR allocat*)) OR RCT) AND (happiness OR happy OR flourish* OR "positive mental health" OR ((subjective OR mental OR positive OR psych* OR personal*) NEAR/3 (well-being OR wellbeing OR wellness OR satisf*)) OR ((life OR domain*) NEAR/3 (satisf* OR evalua* OR appraisal)) OR (positive NEAR/3 (affect* OR emotion* OR mood* OR feeling*)) OR joy*)):ti,ab,kw | 3.045 |
| trialsearch.who.int  (Basic search)  Note: “With results only” was enabled. | (student* OR universit* OR college* OR "higher education" OR campus OR tertiar*) AND (interven* OR practic* OR train* OR activit* OR exercis* OR program* OR promot* OR therap* OR counsel* OR workshop* OR course* OR support) AND ("random* control* trial*" OR "random* trial*" OR "random* assign*" OR "random* allocat*" OR RCT) AND (happiness OR happy OR flourish* OR "positive mental health" OR "subjective well-being" OR "subjective wellbeing" OR "subjective wellness" OR "subjective satisf*" OR "mental well-being" OR "mental wellbeing" OR "mental wellness" OR "mental satisf*" OR "psych* well-being" OR "psych* wellbeing" OR "psych* wellness" OR "psych* satisf*" OR "personal* well-being" OR "personal* wellbeing" OR "personal* wellness" OR "personal* satisf*" OR "life satisf*" OR "life evalua*" OR "life appraisal" OR "satisf* with life" OR "domain satisf*" OR "domain evalua*" OR "domain appraisal" OR "positive affect*" OR "positive emotion*" OR "positive mood*" OR "positive feeling*" OR joy*) | 102 |
| PubMed  (Advanced search, Query box)  Note: joy* not permitted, therefore joy was used. | ((student*[tiab] OR students[mh] OR universit*[tiab] OR college*[tiab] OR "higher education"[tiab] OR campus [tiab] OR teriar*[tiab]) AND (interven*[tiab] OR practic*[tiab] OR train*[tiab] OR activit*[tiab] OR exercis*[tiab] OR program*[tiab] OR promot*[tiab] OR therap*[tiab] OR psychotherapy[mh] OR counsel*[tiab] OR workshop*[tiab] OR course*[tiab] OR support[tiab]) AND ((random*[tiab] AND (trial*[tiab] OR assign*[tiab] OR allocat*[tiab] OR "random allocation"[mh]) OR RCT[tiab]) AND (happiness[tiab] OR happy[tiab] OR flourish*[tiab] OR "positive mental health"[tiab] OR ((subjective[tiab] OR mental[tiab] OR positive[tiab] OR psych*[tiab] OR personal*[tiab]) AND ("well-being"[tiab] OR wellbeing[tiab] OR wellness[tiab] OR satisf*[tiab])) OR ((life[tiab] OR domain*[tiab]) AND (satisf*[tiab] OR evalua*[tiab] OR appraisal[tiab])) OR "personal satisfaction"[mh] OR (positive[tiab] AND (affect*[tiab] OR emotion*[tiab] OR mood*[tiab] OR feeling*[tiab])) OR joy[tiab]))) AND pubmednotmedline[sb] | 1.053 |
