# Supplementary material for: Digitalization of Intervention Delivery and Its Impact on the Effects of Interventions for Mental Well-Being in Higher Education Students: Systematic Review and Meta-Analysis Protocol
Source: JMIR Res Protoc. 2026 Jul 3;15:e88458. doi: 10.2196/88458 (PMC13379693; doi:10.2196/88458)
Supplement: Multimedia Appendix 4 [file resprot_v15i1e88458_app4.docx]

# Appendix 4

Intended structure and content of the data extraction form to be constructed and used in the Covidence software.

| **Data extraction details** | | |
| --- | --- | --- |
| Extractor | *Initials and date* | |
| Verifier | *Initials and date* | |
| Comments/Notes | *Aspects/issues etc. which stands out for this study or this data extraction* | |
| **Study details** | **Extracted data** | **Revised data** |
| Study ID/Record number | *Numeric code for identification* |  |
| Full reference | *AMA 11^th^ Ed.* |  |
| Brief citation | *Author(s) , year of publication* |  |
| Location | *Country/-ies where study has been conducted* |  |
| Study plan | *Protocol or trial registration number and source* |  |
| **Data *from* the study to inform evidence synthesis** | | |
| **Participants** | **Extracted data** | **Revised data** |
| Type/Context/Setting | *Level of education, intervention delivery context* |  |
| Number | *Total N, n per group/condition* |  |
| Age | *Measures of central tendency and dispersion* |  |
| Gender | *Male/Female distribution (n or %)* |  |
| **Intervention** | **Extracted data** | **Revised data** |
| Name | *As presented in study* |  |
| Theme/Category | *As presented in study* |  |
| Duration | *Total intervention time* |  |
| Dose | *Active intervention time* |  |
| Intensity | *Frequency, total and dispersion* |  |
| Mode of delivery | *As presented in study (as much details as possible)* |  |
| **Comparator** | **Extracted data** | **Revised data** |
| Groups | *Number of groups* |  |
| Group characteristics | *Type of groups, n per group* |  |
| **Outcome** | **Extracted data** | **Revised data** |
| Outcome/-s | *Positive affect or life satisfaction or both.* |  |
| Measurement/-s | *Instrument/-s used* |  |
| Baseline | *Mean, St. Dev., n (per group) or other relevant values* |  |
| Post | *Mean, St. Dev., n (per group) or other relevant values* |  |
| Follow-up | *Time, Mean, St. Dev., n (per group) or other relevant values* |  |
| Change, baseline to post | *Mean, St. Dev., n (per group) or other relevant values* |  |
| Change, baseline to follow-up | *Mean, St. Dev., n (per group) or other relevant values* |  |
| Change, post to follow-up | *Time, Mean, St. Dev., n (per group) or other relevant values* |  |
| Interaction effects | *Groups, Times of measure, relevant test statistics* |  |
| **Data *about* the study to inform risk of bias assessment** | | |
| **Randomization process** | **Extracted data** | **Revised data** |
| Allocation sequence generation | *How randomization was conducted* |  |
| Allocation sequence concealment | *How participants/personnel were prevented from knowing* |  |
| Baseline balance | *Notable (not by chance) imbalance(s) in baseline factors* |  |
| **Deviations, intended interventions** | **Extracted data** | **Revised data** |
| Trial protocol changes | *Stated or identified changes from protocol, and causes* |  |
| Blinding | *Participants or personnel blinding procedure* |  |
| Intention-to-treat analysis | *Material basis (participants) for the outcome analysis* |  |
| **Missing outcome data** | **Extracted data** | **Revised data** |
| Outcome data availability | *Missing outcome data per group, proportion* |  |
| Missing data reasons | *As presented in study* |  |
| **Outcome measurement** | **Extracted data** | **Revised data** |
| Appropriateness | *Measurement errors, validity or reliability presented* |  |
| Group differences | *Setting and instruments per group* |  |
| Outcome assessor | *Participant, intervention provider, or observer.* |  |
| Influence on outcome | *Assessor blinding or influence by knowledge of intervention* |  |
| **Reported results** | **Extracted data** | **Revised data** |
| Analysis | *Conformity with pre-specified plan* |  |
| Selection | *All results or selection, reasons.* |  |
