# Supplementary material for: Digitalization of Intervention Delivery and Its Impact on the Effects of Interventions for Mental Well-Being in Higher Education Students: Systematic Review and Meta-Analysis Protocol
Source: JMIR Res Protoc. 2026 Jul 3;15:e88458. doi: 10.2196/88458 (PMC13379693; doi:10.2196/88458)
Supplement: Multimedia Appendix 5 [file resprot_v15i1e88458_app5.docx]

# Appendix 5

| **PICO domain** | **Criteria question** | **Answer/Action** |
| --- | --- | --- |
| Outcome | 1. Was the outcome positive affect or life satisfaction or both? | Yes, proceed. |
|  |  | Maybe, proceed. |
|  |  | No, exclude. |
| Population | 2. Were the study participants students in higher education? | Yes, proceed. |
|  |  | Maybe, proceed. |
|  |  | No, exclude. |
| Intervention | 3. Did the intervention consist of a unified intervention strategy (eg, not multi-intervention-themes program)? | Yes, proceed. |
|  |  | Maybe, proceed. |
|  |  | No, exclude. |
| Comparator | 4. Was it a randomized pre-post intervention-control group study? | Yes, proceed. |
|  |  | Maybe, proceed. |
|  |  | No, exclude. |
| Population | 5. Were the study participants considered to be ‘mentally healthy’? | Yes, proceed. |
|  |  | Maybe, proceed. |
|  |  | No, exclude. |
| Intervention | 6. Was the intervention individually delivered (eg, not group)? | Yes, proceed. |
|  |  | Maybe, proceed. |
|  |  | No, exclude. |

Questions to be used when conducting first stage screening of titles and abstracts.
