# Supplementary material for: Digitalization of Intervention Delivery and Its Impact on the Effects of Interventions for Mental Well-Being in Higher Education Students: Systematic Review and Meta-Analysis Protocol
Source: JMIR Res Protoc. 2026 Jul 3;15:e88458. doi: 10.2196/88458 (PMC13379693; doi:10.2196/88458)
Supplement: Multimedia Appendix 6 [file resprot_v15i1e88458_app6.pdf]

Jag skulle behöva hjälp med att korta ned denna titel: Digitalization of Intervention Delivery and Its Impact on the Effects of Interventions for Mental Well-being in Higher Education Students: Systematic Review and Meta-Analysis Protocol. Förslag?

Thought for 23s

Här är kortare och snärtigare titelförslag (alla på engelska, olika längd/ton men med samma kärna):

Mycket korta

1. Digital Delivery and Student Mental Well-being: Systematic Review & Meta-analysis Protocol
2. Digital Delivery of Student Well-being Interventions: Review & Meta-analysis Protocol
3. Digital Intervention Delivery and Student Well-being: Review & Meta-analysis Protocol

Korta
4) Digital Delivery and Effects of Student Well-being Interventions: Systematic Review and Meta-analysis Protocol
5) Effects of Digital Delivery on Student Well-being Interventions: Systematic Review and Meta-analysis Protocol
6) Delivery Modality and Student Mental Well-being: Systematic Review and Meta-analysis Protocol
7) Digital vs Non-digital Delivery of Student Well-being Interventions: Systematic Review and Meta-analysis Protocol

Mellanlånga (mest formella)
8) Digital Delivery of Mental Well-being Interventions in Higher Education: A Systematic Review and Meta-analysis Protocol
9) Delivery Mode and Outcomes of Student Mental Well-being Interventions: Systematic Review and Meta-analysis Protocol
10) Digitalization and Effectiveness of Student Mental Well-being Interventions: Systematic Review and Meta-analysis Protocol
11) Digital Delivery and Outcomes of Well-being Interventions in Higher Education: Protocol for a Systematic Review and Meta-analysis
12) Intervention Delivery Modality and Student Mental Well-being: Protocol for a Systematic Review and Meta-analysis

Vill du ha svenska varianter också, eller finputsa en av dessa mot en specifik tidskrifts stilguide (t.ex. teckenlängd, kolon/inget kolon, & vs “and”)?
